# Supplementary material for: MetaRibo-Seq measures translation in microbiomes
Source: Nat Commun. 2020 Jun 29;11:3268. doi: 10.1038/s41467-020-17081-z (PMC7324362; doi:10.1038/s41467-020-17081-z)
Supplement: Supplementary file 10 — Supplementary Data 7 [file 41467_2020_17081_MOESM10_ESM.zip › File2/Confidence_VeryHigh_Taxonomy/138581_out.krona.html]

Javascript must be enabled to view this page.

members
magnitude
magnitudeUnassigned
count
unassigned
taxon
rank

138581\_out

10

10
2
superkingdom

phylum
1239
7

species
1262991

SRS048164\_contig\_number\_30447
1

class
186801
6

6
order
186802

3
family
186803

1
841
genus


SRS104693\_contig\_number\_1207
360807
species
1

2
species
39491

SRS017821\_contig\_number\_12581SRS058070\_contig\_number\_28115

family
186806
3

3
1730
genus

1
species

SRS056519\_contig\_number\_11375
142586

2
1897001

SRS018656\_contig\_number\_contig-100\_11335.11335SRS098717\_contig\_number\_contig-100\_12596.65321
species

phylum
1798710
3

3
order
1906119

3
species
1916232

SRS097920\_contig\_number\_31818SRS143148\_contig\_number\_26072SRS148784\_contig\_number\_contig-100\_2.168090
